# Prevalence of intestinal parasitic infections and their associated risk factors among preschool and school children in Egypt

**DOI:** 10.1371/journal.pone.0258037

**Published:** 2021-09-29

**Authors:** Walid Elmonir, Haitham Elaadli, Anan Amer, Hammed El-Sharkawy, Mohamed Bessat, Samy F. Mahmoud, Mustafa Shukry Atta, Wael F. El-Tras

**Affiliations:** 1 Faculty of Veterinary Medicine, Department of Hygiene and Preventive Medicine (Zoonoses), Kafrelsheikh University, Kafrelsheikh, Egypt; 2 Faculty of Veterinary Medicine, Department of Animal Hygiene Zoonoses, Alexandria University, Alexandria, Egypt; 3 Faculty of Medicine, Department of Pediatrics & Neonatology, Tanta University, Tanta, Egypt; 4 Faculty of Veterinary Medicine, Department of Parasitology, Alexandria University, Alexandria, Egypt; 5 Department of Biotechnology, College of Science, Taif University, Taif, Saudi Arabia; 6 Faculty of Veterinary Medicine, Department of Physiology, Kafrelsheikh University, Kafrelsheikh, Egypt; 7 Faculty of Aquatic and Fisheries Sciences (Zoonoses), Kafrelsheikh University, Kafrelsheikh, Egypt; Dokkyo Medical University, JAPAN

## Abstract

Intestinal parasitic infections (IPIs) are among the major public health problems globally, particularly in developing countries like Egypt. This study aimed to evaluate prevalence and risk factors associated with IPIs among preschool and school children in Egypt. A cross-sectional study was conducted on 996 randomly selected preschool and school-aged children in Gharbia governorate during January to April 2018. Stool specimens were examined for the presence of the parasite by direct smear and the formol-ether concentration methods. The overall prevalence of IPIs was 46.2%. *Entamoeba histolytica* and *Ascaris lumbricoides* were the most predominant parasites (12.7% per each). This is followed by *Enterobius vermicularis* (8.6%), *Giardia lamblia* (7.1%), *Cryptosporidium parvum* (1.5%), *Heterophyes heterophyes* (1.4%), *Hymenolepis nana* (0.7%), Hookworms (0.6%), *Fasciola hepatica* (0.5%) and *Dipylidium caninum* (0.4%). Infected children with no symptoms (26.8%) were significantly (*P* < 0.001) more frequent than those with medical complaint (19.4%). Socio-demographic predictors of IPIs were preschool age (OR = 4.9; *P* < 0.001; 95%CI 3.3–7.3), living in rural dwellings (OR = 1.96; *P* < 0.001; 95%CI 1.5–2.5), and belonging to a low-income family (OR = 4.7; *P* < 0.001; 95%CI 2.3–9.3). The absence of safe drinking water, lack of hand washing (after soil contact, or before meals, or after toilet usage), and eating unwashed vegetables were risk factors for IPIs in the study region (OR = 1.3–6.9, *P* < 0.001 –*P* = 0.05). Higher odds for exposure to potential zoonotic parasites were evident in children with pets in their homes for *G*. *lambia* and *D*. *caninum* (OR = 2.1–8.3; *P* = 0.02 –*P* = 0.04), children having household reared ruminants for *C*. *parvum* (OR = 10.4; *P* < 0.001), and children that play with stray animals for *E*. *histolytica* and Hookworm (OR = 1.8–6.3; *P =* 0.04 –*P* = 0.05)compared to other children with no animal contact. The present study highlights the importance of periodic screening and treatment of IPIs in children, deworming companion animals, and public education for effective prevention of IPIs in children in Egypt.

## Introduction

Intestinal parasitic infections (IPIs) represent a significant global public health alert in less developed countries because they result in high morbidity and mortality [1]. They cause over 33% of deaths worldwide [2]. Most diseases caused by the intestinal parasites (worms and protozoa) have been categorized as Neglected Tropical Diseases (NTDs) [3,4] that have been a significant problem in many developing countries. In general, overcrowding, lack of environmental sanitation and safe water, poor hygienic living conditions, severe malnutrition, warm and humid climate, low educational background and lack of personal hygiene are the most potential risk factors for IPIs [5,6].

People of all ages are affected by IPIs; however, Children are the most commonly affected, which is linked to their poor hygienic practices, and weak immune status [7]. In children, IPIs are associated with malabsorption, weight loss, anemia, poor growth rate (stunting), learning difficulties, mental retardation and intellectual problems [8,9]. Overall, millions of preschoolers and school children are at high risk for infection with the protozoan and helminthic parasites [4], requiring effective preventive measures and treatment with deworming drugs at regular periods.

According to estimates of the World Health Organization, approximately 3.5 billion people are exposed to IPIs, and 450 million are diseased as a result; more than half of them are children [10]. There is usually a heavy burden associated with IPIs as it is related with 39 million disability-adjusted life years (DALYs), low economic status, high incidence of diseases, and social inequalities, which consequently lead to financial distress in the family and the community as a whole [11,12].

*A*. *lumbricoides*, *Entamoeba histolytica* and *Giardia lamblia* are common public health problems in the developing countries globally. *A*. *lumbricoides* infects 1.5 billion people worldwide, resulting in an annual morbidity rate of 335 million and 60,000 associated mortalities [9]. *Entamoeba histolytica* is an anaerobic enteric protozoan parasite which is estimated to infect about 50 million people worldwide [13]. Furthermore, *G*. *lamblia* is the most globally common intestinal parasitic protozoan, infecting more than 200 million people worldwide [14].

Analysis of parasitic intestinal infections featured a country-to-country variation in prevalence and predisposing risk factors. In the Middle East, studies on intestinal parasites showed varied prevalence rates; for example, 42.5% in Syria, 33.9% in Qatar, 5.3% in Saudi Arabia, 28.7% in Yemen, 28.5% in Jordan, 74.6% in Palestine, 27.3% in Iran, 17% in Sudan, 12.4% in Lebanon, and 83.1% in Pakistan [15–17]. This difference in prevalence is attributed to many factors such as climatic conditions, geographic area, socioeconomic aspects, personal hygiene and population density [18].

A few studies were conducted in some regions of Egypt, such as El Behera [19], Aswan [20], Assiut [21] and Damietta [22] for identification of the overall prevalence of IPIs in stool specimens of school children. These reports revealed that the majority of IPIs vary from region to region. So, there is a need for periodical assessment of local IPIs to formulate an appropriate control and prevention strategies, especially for the high-risk communities. Therefore, this study was aimed to estimate the current prevalence and associated risk factors of IPIs among preschoolers and school children in Gharbia governorate, Egypt.

## Materials and methods

### Ethical approval

Preliminary permission was requested from the heads of the schools and the health care facilities. Furthermore, the study protocol purpose and importance were explained to parents/custodians of selected children and verbal consents were granted before sampling. In addition, this study was approved by the ethics committee of the Kafrelsheikh University, Egypt, and carried outaccording to the guidelines of the Kafrelsheikh University (Number 2020/1535/010).

### Study location, design and sampling

In this cross-sectional study, the selected population were preschool (1 –<5years old) and school children (5–15 years old) resident in Tanta district of Gharbia governorate, Egypt. Children were classified in two categories: (1) children admitted to hospitals in the study region (Tanta university hospital and one private hospital); (2) Children attending schools in the study region (2 urban schools and two rural schools). Besides age, selection criteria also include children who didn’t take specific anthelmintic, antiprotozoal, purgatives or anti-diarrheal drugs three weeks before the stool sampling. Children with mixed IPIs were excluded. The sample size was estimated with expected prevalence of 30%, 95% confidence interval and, accepted error of 5% using Win episcope 2.0 as a minimum of 323 children. Children were randomly selected form chosen hospitals and schools; only those who fulfilled selection criteria were included in the study. A total of 996 children (235 from hospitals and 761 from schools) were examined between January and April 2018. Two stools samples over ten days were collected from every child. All stool samples were transported to the lab and analyzed immediately after arrival.

Additionally, a structured questionnaire was designed in English then translated into the Arabic language and was piloted with few parents of children. The questionnaire was subsequently revised then administered to all parents/custodians of selected children. The questionnaire included information about sociodemographic characteristics, high risk practices, and other predictors related to zoonotic transmission.

### Stool sample analysis

All stool specimens were examined macroscopically for colour, consistency, blood, mucous, pus, and parasite stages such as adult helminths, larvae, and segments of cestodes, which are visible to unaided eyes. They were then examined by the direct smear using a light microscope and the formol-ether concentration method. For the direct smear method, a drop of emulsified stool sample was put on both ends of the slide, a drop of Lugol’s iodine was added to one stool drop leaving the other drop unstained. Smears were examined first by 100x, and then 400x of Olympus^®^ BX41 optical microscope (Olympus Optical Co., Tokyo, Japan) to detect and identify protozoan and helminth diagnostic stages [23]. The formol-ether concentration method is the gold standard technique for examining IPIs, and thus was applied as previously described [23]. It was prepared by emulsifying 1 gram stool in 8 mL 10% formalin, filtered into another tube, before a 4 mL diethyl ether was added to the filtrate. The mixture was homogeneously mixed (by brief hand mixing) for 1 minute and then centrifuged at 3000 rpm for 1 minute to remove the supernatant. Finally, a small portion of the sediment was examined under the microscope. Well-trained parasitologists examined a minimum of two smears from each stool specimens to detect ova, cysts, trophozoites and larvae of enteric parasites. Similar to the direct smear, 100x and 400x magnifications were applied, with Lugol’s iodine solution was added to smears to facilitate identification of cysts of intestinal protozoan parasites. Moreso, modified Kinyoun’s acid-fast staining (Sigma-Aldrich, St. Louis, Missouri, USA) was done to detect parasites like *Cryptosporidium* [24].

### Data handling and statistical analysis

Univariate regression analysis for the association between the socio-demographic characteristics, high risk practices, potential zoonotic exposure, and the prevalence of detected gastrointestinal parasites in examined children was performed using the SPSS v19 (IBM, Armonk, NY, USA). In all cases, *P* ≤ 0.05 were considered statistically significant.

## Results

Out of 996 examined preschoolers and school children, 460 (46.2%) were found to be infected with intestinal parasites. Ten intestinal parasites were detected in the current study includingprotozoans, nematodes, trematodes and cestodes (Table 1). The overall prevalence of protozoa infections was 21.3%. Three protozoa species were detected including *E*. *histolytica*, *G*. *lamblia*, and *C*. *parvum* in 12.7%, 7.1% and 1.5% of the children, respectively. Gastrointestinal helminthes were detected in 24.9% of examined children. The identified helminthes were *A*. *lumbricoides* (12.7%), *Enterobius vermicularis* (8.6%), *Heterophyes heterophyes* (1.4%), *Hymenolepis nana* (0.7%), Hookworms (0.6%), *Fasciola hepatica* (0.5%) and *Dipylidium caninum* (0.4%) (Table 1).There was no significant difference between protozoan and helminthes infection rates in examined children (*X*^*2*^ = 3.6, *P =* 0.06).

**Table 1:** Prevalence of IPIs and their associated clinical manifestations in children in Gharbia governorate, Egypt.

Parasites		Pos.	Clinical presentation		
			Yes	No	Manifestations
**Protozoa**	**Eh**	126 (12.7)	67 (6.7)	59 (5.9)	Bloat–Nausea–Vomiting–Diarrhea–Loss of appetite
	**Gl**	71 (7.1)	48 (4.8)	23 (2.3)	
	**Cp**	15 (1.5)	9 (0.9)	6 (0.6)	
	**Subtotal**	**212 (21.3)**	**124 (12.4)**	**88 (8.8)**	-
**Helminthes**	**Al**	126 (12.7)	96 (9.6)	30 (3)	Nausea–Vomiting–Abdominal discomfort–Loss of appetite–Weight loss
	**Hh**	14 (1.4)	5 (0.5)	9 (0.9)	
	**Hn**	7 (0.7)	2 (0.2)	5 (0.5)	
	**Hw**	6 (0.6)	4 (0.4)	2 (0.2)	
	**Ev**	86 (8.6)	22 (2.2)	64 (6.4)	Gastrointestinal discomfort–Itchy anus
	**Dc**	4 (0.4)	2 (0.2)	2 (0.2)	
	**Fh**	5 (0.5)	4 (0.4)	1 (0.1)	Extreme abdominal pain–Nausea–Swollen liver–Fever–Jaundice
	**Subtotal**	**248 (24.9)**	**69 (6.9)**	**179 (17.9)**	-
**Total**		**460 (46.2)**	**193 (19.4)**	**267 (26.8)**	-

Eh: Entamoeba histolytica; Gl: Giardia lamblia; Cp: Cryptosporidium parvum; Al: Ascaris lumbricoides; Hh: Heterophyes heterophyes; Hn: Hymenolepis nana; Hw: Hookworms; Ev: Enterobius vermicularis; Dc: Dipylidium caninum; Fh: Fasciola hepatica, Yes: clinical case, No: No clinical symp.

Medical complaints were reported in 19.4% (193/996) of examined children (Table 1). However, infected children with no apparent symptoms (26.8%, 267/996) were significantly more frequent than those with medical complaints (*X*^*2*^ = 15.5, *P* < 0.001). Additionally, protozoa-infected children (12.4%, 124/996) showed clinical signs more frequently than those infected with helminths (6.9%, 69/996) and this difference was significant (*X*^*2*^ = 17.4, *P* < 0.001). Clinical manifestations were recorded as bloat, nausea, vomiting, diarrhea, loss of appetite, and weight loss. In addition to general clinical symptoms, some specific embodiments were documented, such as itchy anus in infections with *E*. *vermicularis* and *D*. *caninum*, while *F*. *hepatica* infections were characterized by fever, jaundice, and swollen liver.

In general, highest IPIs prevalence rates were recorded among children admitted to hospitals (80%), preschool age children (76.9%), male children (48.4%), rural residents (52.8%), children from families with low income (54.3%), and children from parents with medium to high education level (46.8% - 47.3%) (Table 2).

**Table 2:** Prevalence and socio-demographic characteristics of IPIs in children in Gharbia governorate, Egypt.

Variable		No.	Positive Protozoa				Positive Helminthes								Positive Total
			Eh	Gl	Cp	SubT	Al	Ev	Hh	Hn	Hw	Fh	Dc	SubT	
Examined location	Hospital	235	67 (28.5)	48 (20.4)	7 (3)	122 (51.9)	30 (12.8)	22 (9.4)	5 (2.1)	2 (0.9)	4 (1.7)	1 (0.4)	2 (0.9)	66 (28.1)	188 (80)
	School	761	59 (7.8)	23 (3)	8 (1.1)	90 (11.8)	96 (12.6)	64 (8.4)	9 (1.2)	5 (0.7)	2 (0.3)	4 (0.5)	2 (0.3)	182 (23.9)	272 (35.7)
Age	Preschool	160	49 (30.6)	37 (23.1)	2 (1.3)	88 (55)	18 (11.3)	12 (7.5)	3 (1.9)	0 (0)	1 (0.6)	0 (0)	1 (0.6)	35 (21.9)	123 (76.9)
	School	836	77 (9.2)	34 (4.1)	13 (1.6)	124 (14.8)	108 (12.9)	74 (8.9)	11 (1.3)	7 (0.8)	5 (0.6)	5 (0.6)	3 (0.4)	213 (25.5)	337 (40.3)
Sex	Male	463	67 (14.5)	35 (7.6)	5 (1.1)	106 (22.9)	60 (13)	38 (8.2)	5 (1.1)	6 (1.3)	3 (0.7)	3 (0.6)	3 (0.6)	118 (25.5)	224 (48.4)
	Female	533	59 (11.1)	36 (6.8)	10 (1.9)	106 (19.9)	66 (12.4)	48 (9)	9 (1.7)	1 (0.2)	3 (0.6)	2 (0.4)	1 (0.2)	130 (24.4)	236 (44.3)
Residence	Rural	599	86 (14.4)	48 (8)	11 (1.8)	145 (24.2)	100 (16.7)	44 (7.3)	10 (1.7)	6 (1)	3 (0.5)	4 (0.7)	4 (0.7)	171 (28.5)	316 (52.8)
	Urban	397	40 (10.1)	23 (5.8)	4 (1)	67 (16.9)	26 (6.5)	42 (10.6)	4 (1)	1 (0.3)	3 (0.8)	1 (0.3)	0 (0)	77 (19.4)	144 (36.3)
Education level	Low	66	10 (15.2)	4 (6.1)	1 (1.5)	16 (24.2)	6 (9.1)	0 (0)	0 (0)	0 (0)	0 (0)	1 (1.5)	1 (1.5)	8 (12.1)	24 (36.4)
	Medium	799	99 (12.4)	57 (7.1)	12 (1.5)	167 (20.9)	112 (14)	64 (8)	12 (1.5)	7 (0.9)	6 (0.8)	4 (0.5)	2 (0.3)	209 (25.9)	374 (46.8)
	High	131	17 (13)	10 (7.6)	2 (1.5)	29 (22.1)	8 (6.1)	22 (16.8)	2 (1.5)	0 (0)	0 (0)	0 (0)	1 (0.8)	33 (25.2)	62(47.3)
Income level	Low	140	25 (17.9)	13 (9.3)	5 (3.6)	42 (30)	17 (12.1)	7 (5)	3 (2.1)	2 (1.4)	3 (2.1)	1 (0.7)	1 (0.7)	34 (24.3)	76 (54.3)
	Medium	792	100 (12.6)	57 (7.2)	9 (1.1)	167 (21.1)	104 (13.1)	74 (9.3)	11 (1.4)	5 (0.6)	3 (0.4)	4 (0.5)	3 (0.4)	204 (25.8)	371 (46.8)
	High	64	1 (1.6)	1 (1.6)	1 (1.6)	3 (4.7)	5 (7.8)	5 (7.8)	0 (0)	0 (0)	0 (0)	0 (0)	0 (0)	10 (15.6)	13 (20.3)
Total		996	126 (12.7)	71 (7.1)	15 (1.5)	212 (21.3)	126 (12.7)	86 (8.6)	9 (0.9)	7 (0.7)	6 (0.6)	5 (0.5)	4 (0.4)	248 (24.9)	460 (46.2)

Preschool age: 1 - >5 years old; School age: 5–15 years old; Low education: Both Father and Mother have under Secondary School education; High education: Both Father and Mother have over Secondary School education; Intermediate education: between High and Low education; Low Income: <2000 LE/Month; Intermediate Income: Between 2000 to 8000 LE/Month; High Income: >8000 LE/Month.

The association between socio-demographic factors and frequency distribution of detected IPIs were analyzed (Table 3). Higher odds for IPIs were found in children at preschool age (76.9%; OR = 4.9; *P* < 0.001; 95%CI 3.3–7.3), living in rural dwellings (52.8%; OR = 1.96; *P* < 0.001; 95%CI 1.5–2.5) and from family with low income (54.3%; OR = 4.7; *P* < 0.001; 95%CI 2.3–9.3). Same associations were reported for children with protozoa’ infection (OR = 1.6–8.7; *P* < 0.001 –*P* = 0.006). However, for children with helminths infection, rural residence (OR = 1.7; *P =* 0.001; 95%CI 1.2–2.3) was the only significant risk. The children gender and parents’ education level did not affect the prevalence of the IPIs (Table 3).

**Table 3:** Univariate regression analysis for association between the socio-demographic characteristics and prevalence of detected IPIs in examined children in this study.

Variable	Categories	Positive Protozoa				Positive Helminthes				Positive Total			
		%	OR	*P*	95% CI	%	OR	*P*	95% CI	%	OR	*P*	95% CI
**Age**	School	14.8	-	-	-	25.5	-	-	-	40.3	-	-	-
	Preschool	55	7.01	< 0.001	4.9–10.1	21.9	0.8	0.3	0.5–1.2	76.9	4.9	< 0.001	3.3–7.3
**Gender**	Female	19.9	-	-	-	24.4	-	-	-	44.3	-	-	-
	Male	22.9	1.2	0.3	0.9–1.6	25.5	1.1	0.7	0.8–1.4	48.4	1.2	0.2	0.9–1.5
**Residence**	Urban	16.9	-	-	-	19.4	-	-	-	36.3	-	-	-
	Rural	24.2	1.6	0.006	1.1–2.2	28.5	1.7	0.001	1.2–2.3	52.8	1.96	< 0.001	1.5–2.5
**Education level**	High	22.1	-	-	-	25.2	-	-	-	47.3	-	-	-
	Medium	20.9	0.9	0.7	0.6–1.5	25.9	1.04	0.9	0.7–1.6	46.8	0.9	0.9	0.7–1.4
	Low	24.2	1.1	0.7	0.6–2.3	12.1	0.4	0.04	0.2–0.9	36.4	0.6	0.1	0.3–1.2
**Income level**	High	4.7	-	-	-	15.6	-	-	-	20.3	-	-	-
	Medium	21.1	5.4	< 0.001	1.7–17.5	25.8	1.9	0.08	0.9–3.7	46.8	3.5	< 0.001	1.9–6.5
	Low	30	8.7	< 0.001	2.6–29.3	24.3	1.7	0.2	0.8–3.8	54.3	4.7	< 0.001	2.3–9.3

OR: Odd ratio; CI: Confidence interval.

High-risk practices varied according to the type of IPIs (Table 4). Swimming in surface water, lack of hand washing after bathroom use, and eating unwashed vegetables were significantly associated with protozoa infection among examined children (OR = 1.7–5.01; *P* < 0.001 –*P* = 0.04). In contrast, lack of hand washing after soil contact was associated with helminths infection (OR = 1.5; *P =* 0.01). Drinking untreated (e.g. ground) water was associated with protozoa infection (OR = 4.6; *P* < 0.001) (Table 4). All examined high risk practices were significantly associated with the overall IPIs (OR = 1.3–6.9, *P* < 0.001 –*P* = 0.05) except for swimming in surface water (Table 4).

**Table 4:** Univariate regression analysis for association between high risk practices and prevalence of detected IPIs in examined children in this study.

Variable	C.	Positive Protozoa				Positive Helminthes				Positive Total			
		%	OR	*P*	95% CI	%	OR	*P*	95% CI	%	OR	*P*	95% CI
**Drinking water source**	T	18.3	-	-	-	24.1	-	-	-	42.4	-	-	-
	UT	50.5	4.6	< 0.001	2.9–7.1	33	1.6	0.06	0.9–2.5	83.5	6.9	< 0.001	3.9–12.1
**Swimming in surface water**	No	21	-	-	-	25	-	-	-	46	-	-	-
	Yes	57.1	5.01	0.04	1.1–22.5	14.3	0.5	0.5	0.06–4.72	71.4	2.9	0.2	0.6–15.2
**Washing hands after Toilet**	Yes	15.7	-	-	-	24.5	-	-	-	40.2	-	-	-
	No	23.8	1.7	0.004	1.2–2.4	25.1	1.03	0.9	0.8–1.4	46.2	1.4	0.01	1.1–1.9
**Washing hands after soil contact**	Yes	21.8	-	-	-	20	-	-	-	41.8	-	-	-
	No	21	0.9	0.8	0.7–1.3	27.4	1.5	0.01	1.1–2.1	48.4	1.3	0.05	1–1.7
**Washing hands before eating**	Yes	18.5	-	-	-	22.6	-	-	-	41.1	-	-	-
	No	23.1	1.3	0.09	0.9–1.8	26.4	1.2	0.2	0.9–1.7	49.4	1.4	0.01	1.1–1.8
**Washing vegetables before eating**	Yes	18.8	-	-	-	25.1	-	-	-	43.9	-	-	-
	No	48.2	4.03	< 0.001	2.6–6.4	22.4	0.9	0.6	0.5–1.5	70.6	3.1	< 0.001	1.9–4.9

C.: Categories; T: Treated water; UT: Untreated water.

The association between potential zoonotic exposure and prevalence of detected IPIs in examined children was characterized by high odds for overall protozoa infection, *G*. *lamblia*, and *D*. *caninum* in children having pets in their home (OR = 1.7–8.3; *P =* 0.02 –*P* = 0.04) and high odds for *C*. *parvum* in children having household reared ruminants (OR = 10.4; *P* < 0.001). Additionally, exposure to stray animals (dogs and cats), was a risk factor for positive IPIs infection, positive protozoa infection, *E*. *histolytica*, and Hookworms infections among examined children (OR = 1.7–6.3; *P* < 0.001 –*P* = 0.05) (Table 5).

**Table 5:** Univariate regression analysis for association between potential zoonotic exposure and prevalence of detected IPIs in examined children in this study.

Practice	Variable		C.	%	OR	*P*	95% CI
**Keeping animals at home**	Positive Protozoa	Pets	No	20.2	-	-	-
			Yes	30.3	1.7	0.02	1.1–2.7
	*Dipylidium caninum*	Pets	No	0.2	-	-	-
			Yes	1.8	8.3	0.04	1.2–59.3
	*Giardia lamblia*	Pets	No	6.4	-	-	-
			Yes	12.8	2.1	0.02	1.2–3.9
	*Cryptosporidium parvum*	Ruminants	No	1	-	-	-
			Yes	9.2	10.4	< 0.001	3.6–30.3
**Play with stray animals** **(Dogs and cats)**	Positive IPIs		No	45.2	-	-	-
			Yes	58.7	1.7	0.03	1.1–2.8
	Positive Protozoa		No	20.2	-	-	-
			Yes	34.7	2.1	0.004	1.3–3.5
	*Entamoeba histolytica*		No	12.1	-	-	-
			Yes	20	1.8	0.05	1–3.3
	Hookworms		No	0.4	-	-	-
			Yes	2.7	6.3	0.04	1.1–34.9

Other variables (e.g. birds and other parasites) had no significant association.

## Discussion

Epidemiological studies on the prevalence of IPIs in different localities are fundamental to identify high-risk regions and formulate adequate prevention and control interventions [25–27]. This study was carried out to estimate the prevalence of IPIs and their associated risk factors among preschool and school children in the Tanta district of Gharbia governorate, Egypt, from January to April 2018.

Our finding showed that the overall prevalence of IPIs was 46.2%, which was lower than other studies in another location in Egypt (56.3%) [21] and in Ethiopia (62.4%) [28]. Yet, our finding was higher than other studies in some Egyptian governorates such as Damietta in lower Egypt (30.7%) [22] and Aswan in Upper Egypt (31%) [20]. Additionally, our IPIs prevalence rate was higher than studies in other countries as Ghana (15%) [29], and Saudi Arabia (5.3%) [17].

Out of ten parasitic infections detected in the current study, *A*. *lumbricoides* (12.7%), and *E*. *histolytica* (12.7%) were the most prevalent parasites. Both parasites account for nearly half of the infection cases (54.8%, 252/460). Similarly, high rates of *A*. *lumbricoides* (11.4% and 22.7%), *and E*. *histolytica* (8.1% and 8.1%), were previously recorded in Egypt [21] and Ethiopia [28], respectively. In contrast, low rates (0%–1%) of theses parasites were reported in Ghana [29]. This variation in the prevalence rates between countries or even in the same country could be attributed to different factors such as various climatic factors, environmental hygiene, socio-economic conditions and the application of precautionary control measures and study methodology [30].

Clinical manifestations were reported in 19.4% (193/996) of examined children. Majority of signs were nonspecific abdominal discomfort signs such as nausea, vomiting, diarrhea, loss of appetite, and weight loss. However, in few instances, parasite-specific clinical symptoms were also reported as itchy anus in case of *E*. *vermicularis* and *D*. *caninum* infections, or jaundice associated with fever and swollen liver caused by *F*. *hepatica* cases. Nevertheless, asymptomatic children with IPIs (26.8%, 267/996) were significantly more frequent than symptomatic children (*P* < 0.001). This was in agreement with previous studies [17,31] and may be attributed to high rate of carrier state or low parasite burden that usually go unnoticed and untreated [31]. ’It should be noted that clinical manifestations among examined children were more frequently associated with protozoa than helminthes infections (*P* < 0.001). This agreed with Liao et al. [32] who reported a positive correlation between gastrointestinal manifestations and protozoa infections as *E*. *histolytica* or *G*. *intestinalis*, yet these signs didn’t correlate with helminth infections. The absence of a clear correlation between clinical picture (in the form of specific symptoms) and occurrence of IPIs is one of the leading causes of IPIs burden and prevalence underestimation as many infected children don’t seek medical care for treatment. This highlights the importance of routine surveillance to detect carrier and asymptomatic cases for timely therapeutic interventions and disease eradication especially in highly endemic developing countries as Egypt.

Several Socio-demographic variables as age, residence, family income and others were previously recorded to predict IPIs among children worldwide [17,28,29,33]. In this study, IPIs were seven times more likely to occur in preschool than school-age children (*P* < 0.001). This could be due to better understanding and application of personal hygiene measures in the older group, as previously recorded [29]. The rural residence was significantly associated with IPIs in examined children (*P* 0.006), which agreed with previous reports from developing countries [28,33]. This could be attributed to several factors as poor sanitation of the rural environment, low access to clean drinking water, inadequate sewer drainage, and high rate of animal contact; all these factors were previously recorded as risk factors for IPIs (17, 28, 29]. Also, rates of IPIs were significantly lower in children belonging to families with high income than those in the middle to low-income families (*P* < 0.001), which was in line with [19]. This could be attributed to improved environmental sanitation, efficient food intake, better medical care and a consequent better immune response; these factors may affect the prevalence of IPIs in high-income families.

There was no significant association between children gender and the occurrence of IPIs in this study. This agrees with previous reports [17], but differs from another study that showed higher IPIs rates among male children [34]. The recent increase of female children education and their access to schools as male children decreased the gap in exposure frequencies related to gender and may explain why our results differ from previous studies. Additionally, parents’ educational levels didn’t affect the rates of their children IPIs in this study, which was in line with studies elsewhere [17,28]. This may highlight that formal education doesn’t provide adequate health education especially with regard to disease prevention.

We investigated six of high-risk practices for IPIs in children in this study. Five of these practices were significantly associated with IPIs in children in the study region. These practices included the absence of safe drinking water, eating unwashed vegetables, and lack of hand washing after soil contact, or before meals or after bathroom usage (OR = 1.3–6.9, *P* < 0.001 –*P* = 0.05). Some or all of these practices were predictors of IPIs among children in several studies in Egypt and elsewhere [17,21,28]. The effect of some practices depended on the type of IPIs. For example, swimming in surface water was associated with protozoa infection (OR = 5.01, *P =* 0.04) and lack of hands washing after soil contact was linked to helminths infection (OR = 1.5, *P =* 0.01). This was expected since many protozoa are transmitted through water-borne infection (e.g. *E*. *histolytica* and *G*. *lamblia*), and helminths are soil associated infections (e.g. *A*. *lumbricoides* and Hookworms) as previously reported [17,28,35].

Pets and other animals are natural reservoirs for many parasites. Nine of 10 detected IPIs in this study are zoonotic, and they were previously reported in several animals’ surveys in Egypt [35]. Presence of pets or ruminants in the household was significantly (*P* < 0.001 –*P* = 0.02) associated with children IPIs in the current study. This was in agreement with other studies in Egypt [35,36] and elsewhere [29]. Owning a pet was significantly (OR = 1.7, *P* = 0.02) associated with protozoa infection, especially *G*. *lamblia* (OR 2.1, *P* 0.02) in children. Dogs and cats are known reservoirs of many zoonotic protozoa including *G*. *lamblia* [35,37] and zoonotic transmission between these animals and humans are reported in several studies in Egypt [35]. *Cryptosporidium parvum* prevalence in children was strongly associated with proximity to domesticated ruminants (OR = 10.4, *P* < 0.001). The previous studies that recorded high detection frequency of same zoonotic subtype of *C*. *parvum* in cattle and humans [36,38], and close association between humans infection and ruminants contact in Egypt [36], support our findings. *Dipylidium caninum*, is a zoonotic cestode transmitted by dogs and cats. Children dipylidiasis was significantly associated with having a pet at home (OR = 8.3, *P =* 0.04), which agreed with several previous reports worldwide [39]. This is the first report of children dipylidiasis cases in Egypt, yet *D*. *caninum* was previously detected in dogs and cats in Egypt [35,37]. Playing with stray animals (dogs and cats) was associated with children IPIs (OR = 2.1, *P =* 0.004) especially protozoa infection (OR = 8.3, *P =* 0.04). Several reports worldwide highlighted the role of stray animals as reservoir and source for human infection with zoonotic parasites [35,37,40]. For individual parasites, *E*. *histolytica* (OR = 1.8, *P =* 0.05), and Hookworms (OR = 6.3, *P =* 0.04) infections were positively associated with stray animal contact. Hookworms infection in humans is caused by *Ancylostoma* spp. Dogs and cats are reservoirs of zoonotic nematode spp. as *A*. *caninum* and *A*. *ceylanicum* known to invade and parasitize humans skin and intestine [40]. Transmission of hookworms infection is usually associated with bare skin contact with contaminated soil containing third stage larva and, in few instances, hand to mouth infection with contaminated hand [28,30,35,40]. It is not clear how stray animal contact contribute to hookworm infection in children in this study. It is however, opined that children caught the infection by contaminating their hands with dirt that covers coat of stray animals or their surrounding. In support to this theory, hookworm infection was significantly associated with lack of hand wash and soil contact activity as waste disposal by burying in previous studies [28,30].

## Conclusion

Our study highlighted high rates of IPIs among children in the study region. *E*. *histolytica* and *A*. *lumbricoides* were the most prevalent parasites. The important risk factors associated with IPIs are preschool age, rural residence, low family’s income, shortage of safe water, lack of hand washing (before meals, after soil contact and after toilet use), eating raw or unwashed vegetables, keeping pets or ruminants at household, and close contact with stray animals. Therefore, it is highly recommended to pursue periodic screening and treatment of IPIs in children, deworming of domestic or companion animals, and public health education about risks and prevention of IPIs especially concerning stray animal contact and personal hygiene. These measures may help to prevent or reduce the prevalence and the risks of IPIs in the study region.

## Supporting information

S1 File (PDF)
