# Supplementary material for: Prevalence of intestinal parasitic infections and their associated risk factors among preschool and school children in Egypt
Source: PLoS One. 2021 Sep 29;16(9):e0258037. doi: 10.1371/journal.pone.0258037 (PMC8480785; doi:10.1371/journal.pone.0258037)
Supplement: S1 File — (PDF) [file pone.0258037.s001.pdf]

Date:…………. Location ID:………… Participant ID:…………

Questionnaire

Our aim for conducting this questionnaire is to identify the risk factors associated with Intestinal parasitic infections (IPIs) among Preschool and School Children in Egypt. This would help in planning effective preventive measures for such diseases and avoiding their potential health hazards among children in Egypt. Participants personal data obtained in this questionnaire are confidential.

(A) Identification:

1. Participant's name (optional): ……………………………..………………..……………

2. Contact Phone: ……………………………………..…………..……………………

3. Address:……………………..……………………………………..…………………………….

(B) Socio-demographic characteristics:

4. How old is the child?

- Preschool age (1 – >5 years old) ☐
- School age (5 – 15 years old) ☐

5. What is the gender of the child?

- Male ☐
- Female ☐

6. Where is the child permanent residence?

- Rural (Village) ☐
- Urban (City) ☐

7. What is the child's father education level?

- Less than Secondary School education ☐
- Secondary School education (or equivalent) ☐
- Higher than Secondary School education (University or equivalent) ☐

8. What is the child's mother education level?
   - Less than Secondary School education ☐
   - Secondary School education (or equivalent) ☐
   - Higher than Secondary School education (University or equivalent) ☐

9. What is the child's family income level?
   - Low (< 2000 LE / Month) ☐
   - Medium (2000 - 8000 LE / Month) ☐
   - High (> 8000 LE / Month) ☐

(C) High risk practices associated with IPIs:

10. What is the source of drinking water at child home?
    - Treated water (Tap water) ☐
    - Untreated water (Well water) ☐

11. Does child swim/play in surface water (e.g. water canals)?
    - Yes ☐
    - No ☐

12. Does child wash hand before eating?
    - Yes ☐
    - No ☐

13. Does child wash hand after Toilet?
    - Yes ☐
    - No ☐

14. Does child wash hand after contact or play with soil?
    - Yes ☐
    - No ☐

15. Does child wash vegetables and fruits before eating them?

- Yes ☐
- No ☐

(D) Animal contact (potential zoonotic exposure):

16. Are there any animals reared at child home?

- Yes ☐
- No ☐

17. If Yes, What is the kind of these animals?

- Ruminants (cow, buffalo, sheep, goat) ☐
- Poultry (chicken, turkey, duck, geese) ☐
- Pets (dog, cat) ☐

18. Does child contact or play with stray animals (dogs, cats)?

- Yes ☐
- No ☐

(E) Clinical presentation of the participant child:

19. Does the child currently suffer any signs of illness?

- Yes ☐
- No ☐

20. If Yes, What are these signs?

……………………………………………………………………………………………
